# Comparative Analysis of *Phlebotomus argentipes* Vector of Leishmaniasis in India and Sri Lanka

**DOI:** 10.3390/microorganisms12071459

**Published:** 2024-07-18

**Authors:** Sachee Bhanu Piyasiri, P.A. Fathima, Harish Kumar Shah, Sanath Senanyake, Nilakshi Samaranayake, Nadira Darshani Karunaweera, Manju Rahi, Prasanta Saini

**Affiliations:** 1 Department of Parasitology, Faculty of Medicine, University of Colombo, Colombo 0800, Sri Lanka; sachee.parasit@stu.cmb.ac.lk (S.B.P.); sanath@parasit.cmb.ac.lk (S.S.); nilakshi@parasit.cmb.ac.lk (N.S.); nadira@parasit.cmb.ac.lk (N.D.K.); 2 Vector Control Research Centre, Field Station, Kottayam 686003, Kerala, Indiaharishkumar883@gmail.com (H.K.S.); drmanjurahi@gmail.com (M.R.)

**Keywords:** *Phlebotomus argentipes*, sandfly, salivary proteins, morphometrics, phylogenetic analysis, Sri Lanka, India, *Leishmania donovani*

## Abstract

*Phlebotomus argentipes* is the predominant sandfly vector of leishmaniasis in the Indian subcontinent. India and Sri Lanka primarily report visceral and cutaneous leishmaniasis caused by *Leishmania donovani*. We compared *Ph. argentipes* from two locations, focusing on its morphological, molecular, and salivary protein characteristics. Sandflies were captured using CDC light traps and cattle-baited net traps. Species identification and morphological comparisons were carried out using standard taxonomic keys. DNA extracted from 12 Sri Lankan sandfly samples was PCR-amplified and sequenced for the variable region of Cytochrome oxidase subunit I. Existing DNA sequences of India from GenBank were utilized for a phylogenetic analysis between Sri Lanka and India. Salivary protein profiles were studied using SDS-PAGE, Western blot, and electrospray ionization/LC/MS/MS. The morphological similarities observed between female *Ph. argentipes* from India and Sri Lanka suggest the presence of *Ph. argentipes* var. *glaucus*. A phylogenetic analysis showed genetic divergence between *Ph. argentipes* populations, but both shared a similar salivary protein profile. A common, strong 30 kDa immunogenic band comprised PagSP05, PagSP06, and PagSP17 proteins of *Ph. argentipes*. The similarity between the immunogenic salivary proteins suggests their potential use as common markers for vector exposure or immune response stimulants across regions. The use of multiple samples for each category of serum would improve the comprehensiveness of the immunogenic profiles obtained.

## 1. Introduction

Leishmaniasis is a vector-borne illness caused by protozoan parasites belonging to the genus *Leishmania*. It manifests in various clinical forms, including cutaneous leishmaniasis (CL), muco-cutaneous leishmaniasis (MCL), and visceral leishmaniasis (VL). Currently, over 1 billion individuals reside in regions endemic for leishmaniasis, placing them at risk of infection. Approximately 30,000 new cases of VL and over 1 million new cases of CL are reported each year [1].

Sandflies, comprising approximately 800 known species globally, play a crucial role in the transmission of *Leishmania* parasites [2,3]. Among these species, more than 40 species are suspected or proven vectors of *Leishmania* [2,4]. VL, commonly known as kala-azar in the Indian subcontinent (ISC), is a significant public health issue in Bangladesh, India, and Nepal [5,6]. It is caused by *Leishmania donovani*, which is transmitted by the sandfly *Phlebotomus argentipes* [7,8,9]. In India, VL is primarily caused by *L. donovani* [10], with some new sites being identified where CL caused by *L. donovani* is emerging, raising significant concerns [11,12,13,14] about atypical parasite–phenotype combinations. However, Sri Lanka has reported a similar atypical occurrence of localized CL caused by *L. donovani* [15,16] since 1992 [17], transmitted by *Ph. argentipes*.

*Ph. argentipes* is known to comprise three complex members [18,19] and exists in two morphological species (morphospecies A and B) in South India and Sri Lanka [19]. It has been suggested that this species consists of sibling species with varying vectorial capacities [19]. Understanding the biology of *Ph. argentipes* is essential for identifying vector dynamics and comprehending the observed differences in disease phenotypes within the region. A vital aspect of sandflies’ vectorial capacity lies in the composition of their saliva [20,21,22], which exhibits variations not only among different species [23,24] but also within populations originating from distinct geographical areas [21,25,26,27]. This variability is a critical factor in understanding the interaction between sandflies and *Leishmania* parasites, as well as in identifying salivary protein-based biomarkers of exposure and vaccine candidates.

Given the observed clinical distinctions in *L. donovani*-induced phenotypes between India and Sri Lanka, these variations may influence the interaction between the parasite and the vector, subsequently impacting infection dynamics. This study aimed to comprehensively examine the variations and similarities in the vector *Ph. argentipes* in India and Sri Lanka, which serves as the primary transmitter of *L. donovani*, particularly focusing on its morphological, molecular, and salivary immunogenic proteins.

## 2. Materials and Methods

### 2.1. Sandfly Collection

**Figure 1:**
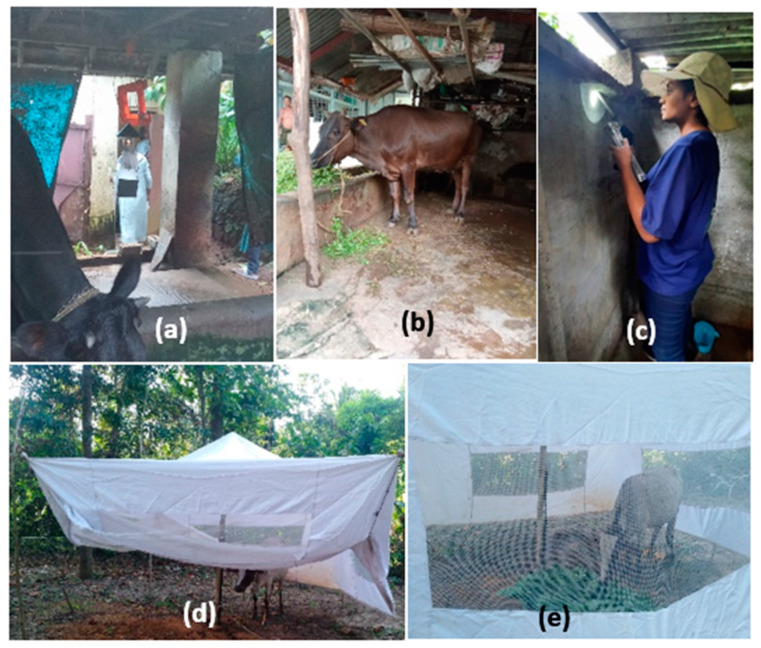
Sandfly collection habitats. (**a**,**b**) CDC-modified light trap collection near cattle sheds in India. (**c**) Interior collections at outdoor bathrooms by mechanical aspirators in India. (**d**,**e**) Cattle-baited net trap collection in Sri Lanka.

Sandflies were collected from the Kottayam, Thrissur, and Kollam districts of Kerala, India, from October to December 2023. The majority of these collection sites were characterized by wet vegetative land cover, with an average annual temperature of approximately 28.5 °C and average annual rainfall of around 2500 mm. Vegetation in the collection areas includes dense forests, rubber plantations, and coconut groves. The proximity to water bodies such as rivers and ponds supports a diverse ecosystem. Human activities in these areas are predominantly agricultural, with a focus on rubber and coconut cultivation. The local population density is moderate, with scattered settlements and small villages. Livestock, particularly cattle and goats, are commonly found near human dwellings. The specimens were captured using CDC-modified light traps (set up from 6 p.m. to 6 a.m.) and mechanical aspirators (the collections were conducted in daytime from 7 a.m. to 10 a.m. in the morning) deployed in proximity to cattle sheds and outdoor settings, such as the interiors of bathrooms (Figure 1). In Sri Lanka, sandflies were captured in Ambalantota, located in the Southern Province, which is recognized as an endemic hotspot for CL infection [28]. The geography of this area is similar to Indian sites, with a mean average temperature of around 27 °C and an annual average rainfall ranging from 1250 mm to 2000 mm. The vegetation includes mixed agricultural fields, home gardens, and patches of forest. The primary agriculture is paddy cultivation, supported by an extensive system of irrigation canals. The human population density in these areas is relatively low, with communities engaged mainly in farming and small-scale trade. Livestock, including cattle and poultry, are common in these settings. Collections were made using cattle-baited net traps (CBNTs) during January to August 2022 and the collections were conducted during 10 p.m. to 3 a.m. (Figure 1). Sandfly collections were conducted using standardized protocols to ensure consistency across different sites and by a trained field team to minimize variability. Live sandflies were subsequently transported to the laboratory for further analysis.

### 2.2. Morphological Identification of Sandflies

The captured sandflies were sorted up to genus level under dissection microscope, mounted in Hoyer’s medium according to the standard protocol, and examined under a compound light microscope (Olympus, Tokyo, Japan). Species identification was carried out utilizing publication by Ilango, 2010 [19], and standard taxonomic keys by Lewis 1978, and Kalra and Bang 1983 [29,30]. 

### 2.3. DNA Extraction, PCR Amplification, and Sequencing

DNA was extracted from 12 samples (8 females and 4 males) from Sri Lanka using the DNeasy Blood and Tissue kit (QIAGEN, Hilden, Germany). Abdominal, thoracic, and leg portions were used for DNA extraction. Negative controls were included in DNA extractions to detect potential contamination. PCR amplification of cytochrome oxidase subunit I gene (cox 1) was conducted with primers COF (5′-GGTCAACAAATCATAAAGATATTGG-3′) and COR (5′-TAAACTTCAGGGTGACCAAAAAATCA-3′) following the protocol described by Kumar et al., 2012 [31]. The PCR run included positive controls (known sandfly DNA) and a negative control. PCR products were electrophoresed on a 1.5% agarose gel. Gel bands (720 bp) were excised and purified products were sent for custom sequencing. 

Sandfly samples were collected from both Kerala, India, and Sri Lanka. We prioritized sequencing the Sri Lankan samples, as this study represents the first instance of DNA sequencing for the study site. The Indian sequences (Kerala and Bihar) from GenBank were then utilized in our phylogenetic analysis, enabling a robust comparison between the populations from India and Sri Lanka.

### 2.4. Sequence Alignment and Phylogenetic Analysis

Sequencing reactions included quality control steps to verify the accuracy of the sequences obtained. DNA sequences were edited and visualized using Chromas 2.6.6. The sequences were then aligned and contigs were prepared using MEGA 7. The sequences were subjected to a BLAST search on the National Center for Biotechnology Information (NCBI). Phylogenetic analysis was conducted using MEGA 7 (http://www.megasoftware.net/ (accessed on 30 December 2023)). The analysis included 12 *Ph. argentipes* sequences from Sri Lanka, as well as 12 sequences obtained from NCBI for Kerala, India and 12 sequences from Bihar, India, which is considered a VL hotspot. The neighbor-joining method was employed with 1000 bootstrap replicates and the Kimura-2 parameter distance model. The accession numbers (PP064129, PP064130, PP064131, PP064132, PP064133, PP064134, PP064135, PP064136, PP064137, PP064138, PP064139, and PP064140) present in this study were submitted to GenBank.

### 2.5. Salivary Gland Dissection

Dissections were performed using standardized protocols to ensure consistency across all samples. Female *Ph. argentipes* sandflies captured in the wild were provided with a 30% sucrose solution for one day and then immobilized at −20 °C for 10 min. Subsequently, they were dissected on a glass slide in cold phosphate-buffered saline (PBS) with a pH of 7.4, using fine needles and a dissection microscope. The salivary glands were individually placed in a PCR tube containing 1 μL of 1× PBS and stored at −80 °C until further analysis. Upon the dissection of salivary glands, the morphology of the glands was observed under a compound light microscope. 

### 2.6. Salivary Gland Homogenate (SGH) Preparation

The salivary glands of *Ph. argentipes* from two countries were dissected in 20 pairs and suspended in 20 µL PBS separately. They were subjected to rapid freezing in liquid nitrogen for 2 min, followed by thawing in a 37 °C hot water bath for 1 min. After thawing, the samples were centrifuged at 14,000 rpm for 1 min, resulting in a pellet. This pellet was then sonicated for 2 min and subjected to another centrifugation at 8000 rpm for 2 min. The resulting supernatant was meticulously separated from the pellet and used for further investigations. Homogenates were prepared under consistent conditions, including their temperature and buffer composition.

### 2.7. Electrophoretic Separation of Salivary Proteins

The comparison of salivary proteins of Indian and Sri Lankan *Ph. argentipes* was assessed through SDS-PAGE. In this method, SGH from a previous step (equivalent to 40 µg of total salivary proteins per lane were separated on a 12% polyacrylamide gel under non-reducing conditions using an SDS-PAGE apparatus (Bio Rad-Mini Protein Tetra Cell, Hercules, CA, USA). Each sample was run in duplicate to verify the reproducibility of the band patterns. The separated protein bands were stained with Coomassie Blue G-250 (Sigma-Aldrich, St. Louis, MI, USA). Subsequently, the stained gel was destained using a solution containing 40% methanol and 20% acetic acid to observe the protein bands.

### 2.8. Immunogenic Proteins 

SGH proteins of *Ph. argentipes* from India and Sri Lanka (40 µg of protein per lane) were electrophoretically separated on a 12% polyacrylamide gel under non-reducing conditions (Bio Rad-Trans Blot, Hercules, CA, USA). The separated proteins were transferred onto an activated Polyvinylidene Fluoride (PVDF) membrane (Immobilon-P, Dublin, Ireland) and subsequently cut into strips. These strips were blocked in 5% non-fat milk diluted in Tris-buffered saline with 0.05% Tween 20 (TBS-Tw) overnight at 4 °C, followed by a 3 h incubation with human sera. Sera were obtained from a fresh VL patient, a fresh CL patient (those who had not received treatments yet), a healthy endemic individual (EN), and a healthy individual living in a non-endemic area of leishmaniasis (NE) as a negative control. VL, CL, and EN individuals were selected from study sites in India and Sri Lanka separately for the test, and they had not traveled to other regions or countries in the past year. All the individuals were between 18 years and 60 years. The sera were diluted at a ratio of 1:80 in TBS-Tw. After a washing step with TBS-Tw, the strips were incubated for 1 h at room temperature with peroxidase-conjugated goat anti-human IgG antibody (Sigma-Aldrich, USA), diluted at a ratio of 1:5000 in TBS-Tween. The chromogenic reaction was developed using TMB or DAB substrate solutions. Following the reaction, the membrane strips were rinsed with distilled water. A molecular-weight protein marker (clearly stained protein marker by Takara Bio, San Jose, CA, USA) was employed to estimate the sizes of the immunogenic protein bands.

### 2.9. Identification of Highly Expressed Immunogenic Salivary Proteins 

The ~30 kDa band, which represents a common antigenic salivary protein identified in Western blot, was isolated from the SDS-PAGE gel and preserved in 50% methanol. Samples were prepared using standardized protocols to ensure consistency in protein digestion and peptide extraction. This sample was subjected to Electrospray Ionization/Liquid Chromatography/Tandem Mass Spectrometry (ESI/LC/MS/MS) for protein identification, comparing against UniProt databases. A proteomics data analysis was conducted using Progenesis QI for Proteomics Version 4.2 software.

## 3. Results

### 3.1. Sandflies of Field Collections

**Figure 2:**
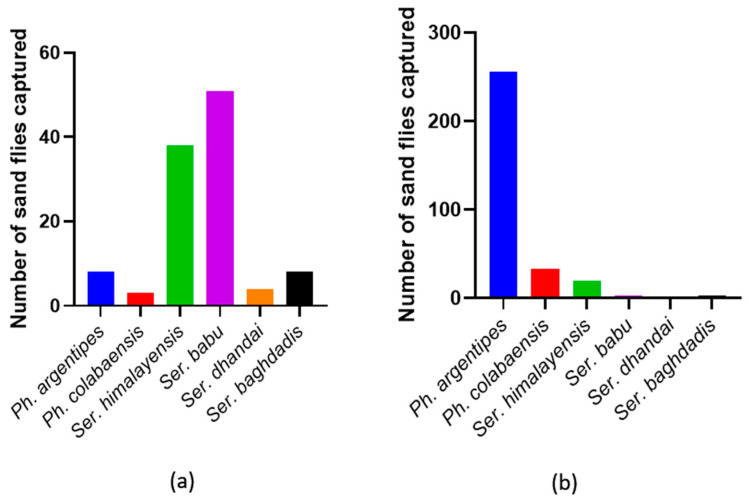
Sandfly species captured during the field collections in India. (**a**) Species composition of resting sandfly collection in outdoor bathrooms. (**b**) Species composition of sandflies collected in cattle sheds.

In total, 426 sandflies were collected from various sites in Kerala, India, and they exhibited a mixed population. The predominant species, *Ph. argentipes*, constituted a large majority (81.53%, n = 256/314) of the collected specimens, primarily found in cattle shed collections. Conversely, the majority of Ser. babu flies (45.54%, n = 51/112) were collected in outdoor bathrooms. Other identified species included *Ph. colabaensis*, *Ser. himalayensis*, *Ser. dhandai*, and *Ser. baghdadis* (Figure 2 and Table S1). In Sri Lanka, all the sandflies collected (n = 389) from Ambalantota were *Ph. argentipes*.

### 3.2. Morphology of Indian and Sri Lankan Ph. argentipes

**Figure 3:**
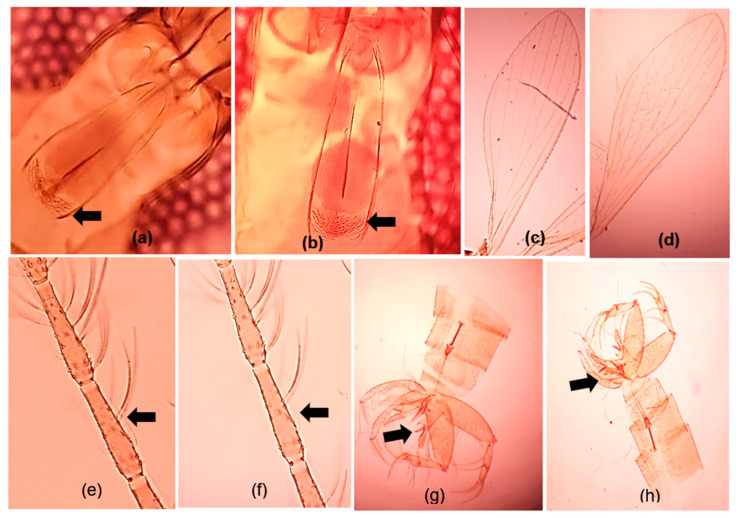
Morphological characteristic features of *Phlebotomus argentipes* flies, under compound light microscope: (**a**,**b**) cibarium of Indian and Sri Lankan female *Ph. argentipes,* respectively (×10×40); (**c**,**d**) wing geometry of Indian and Sri Lankan *Ph. argentipes,* respectively (×10×10); (**e**,**f**) sensilla chaeticae on second antennal flagellomere of Indian and Sri Lankan female *Ph. argentipes,* respectively (×10×40); (**g**,**h**) three-lobed paramere of Indian and Sri Lankan male *Ph. argentipes,* respectively (×10×10).

The wings of both species exhibited a broad width and asymmetry, particularly from top to bottom, with a wing overlap (R1 overlap with R2/complete length of R2) of approximately 0.2. The absence of armament or the presence of dispersed spicules and the lack of a pigment patch in the cibarium were evident in both species (Figure 3). Both species shared this characteristic. The wing index (R2/R2 + 3) was approximately 2. The ratio of sensilla chaetica lengths to antennal flagellomere exceeded 0.5 for both female species, indicating a similarity between specimens identified in India and Sri Lanka (Figure 3e,f). According to the standard taxonomic keys and taxonomic studies [19,29,30], these specimens were identified as *Ph. argentipes* var. *glaucus*. Further, male specimens were distinctive in having a pair of spines lying parallel to the aedegus, and a paramere with three unequal lobes (Figure 3g,h).

### 3.3. Phylogenetic Analysis of Indian and Sri Lankan Ph. argentipes

A set of 12 *cox1* sequences of *Ph. argentipes* specimens collected from Ambalantota, Sri Lanka, (Figure S1; accession numbers: PP064129, PP064130, PP064131, PP064132, PP064133, PP064134, PP064135, PP064136, PP064137, PP064138, PP064139, and PP064140) were utilized for the phylogenetic analysis. The alignment of these *cox1* sequences with those existing in the NCBI public domain validated their identification as *Ph. argentipes* for the *cox1* gene.

**Figure 4:**
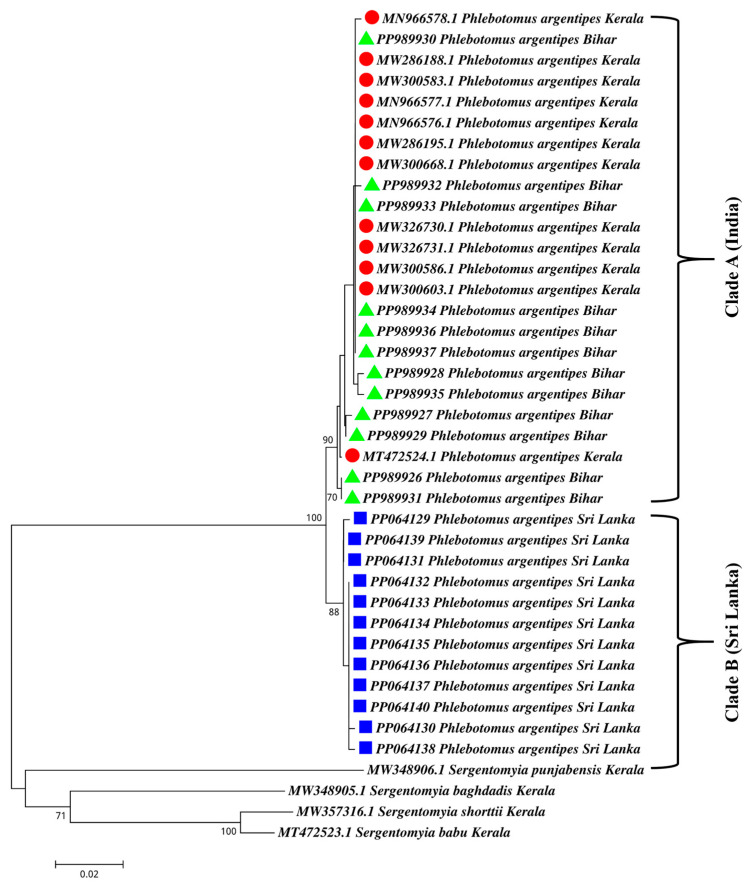
Phylogenetic analysis using the Neighbour Joining method using the *cox1* sequences of *Phlebotomus argentipes* populations from two countries; Ambalantota, Sri Lanka (blue squares), India: Kerala (red circles), and Bihar (green triangles). *Sergentomyia punjabensis*, *Ser. shortii, Ser. baghdadis,* and *Ser. babu* were selected as outgroup members.

The phylogenetic analysis utilized the 12 sequences from Sri Lanka and another 12 sequences from Kerala, India, and 12 sequences from Bihar, India, obtained from GenBank. We observed nine synonymous mutations in the DNA sequences of the *cox1* gene of Sri Lankan *Ph. argentipes* compared to the Indian sequences. The neighbor-joining results are shown in Figure 4. Sandflies originating from clade A clustered with *Ph. argentipes* from India, while those in clade B were from Sri Lanka (Figure 4). In the phylogenetic tree, a mixture of clades was observed, with seven lineages in clade A among *Ph. argentipes* sandfly sequences from Kerala, India, and Bihar, India, and with three lineages among Sri Lankan sequences in clade B. This observation suggests genetic divergence between sandfly populations from Sri Lanka and India. Despite this divergence, both groups shared a recent common ancestor (Figure 4).

### 3.4. Morphology of Salivary Glands 

**Figure 5:**
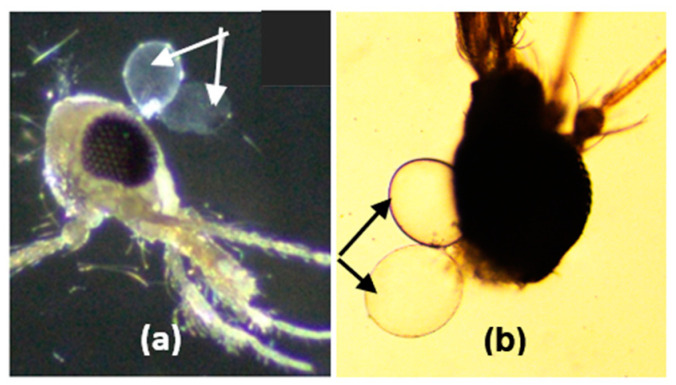
Morphology of salivary glands (marked with arrows) of *Phlebotomus argentipes* under compound light microscope; (**a**) salivary gland of Indian *Ph. argentipes* (×10×40); (**b**) salivary gland of Sri Lankan *Ph. argentipes* (×10×40).

Both species of female *Ph. argentipes* in Kerala, India, and Ambalantota, Sri Lanka, exhibited heterogeneous salivary glands (Figure 5) and the size of the two lobes varied according to the age of the fly.

### 3.5. Salivary Gland Protein Profiles

**Figure 6:**
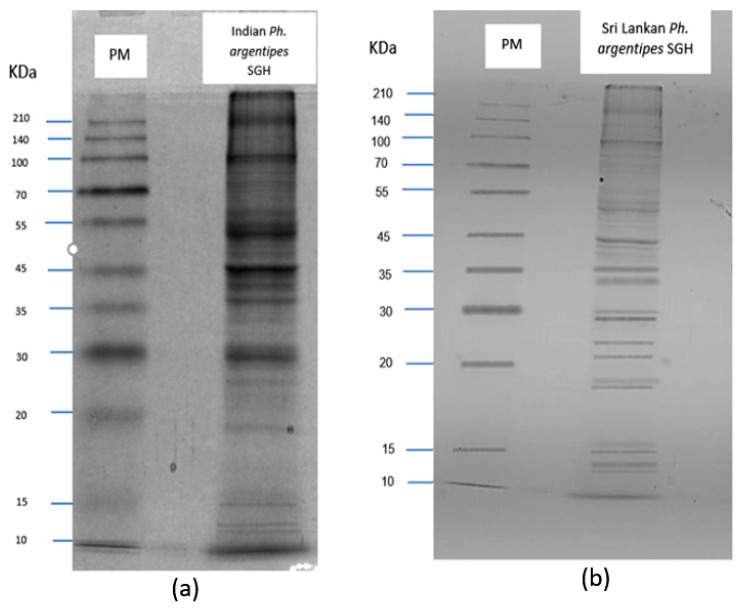
Salivary gland protein profile of *Phlebotomus argentipes*. Staining with Coomassie Blue on a 12% Sodium dodecyl sulfate polyacrylamide gel (SDS-PAGE). (**a**) Salivary gland homogenate (SGH) of Indian *Ph. argentipes;* (**b**) SGH of Sri Lankan *Ph. argentipes*. PM; protein molecular weight marker.

In the Indian *Ph. argentipes*, several prominent protein bands were observed, particularly around ~15 kDa, ~30 kDa, ~35 kDa, ~40 kDa, ~45 kDa, and ~50 kDa (Figure 6a). These bands demonstrated either similar or minimal expression in the Sri Lankan *Ph. argentipes*, with notable differences observed in bands around ~30 kDa, >35 kDa, ~35 kDa, ~45 KDa, and ~50 kDa (Figure 6b).

### 3.6. Immunogenic Salivary Proteins

**Figure 7:**
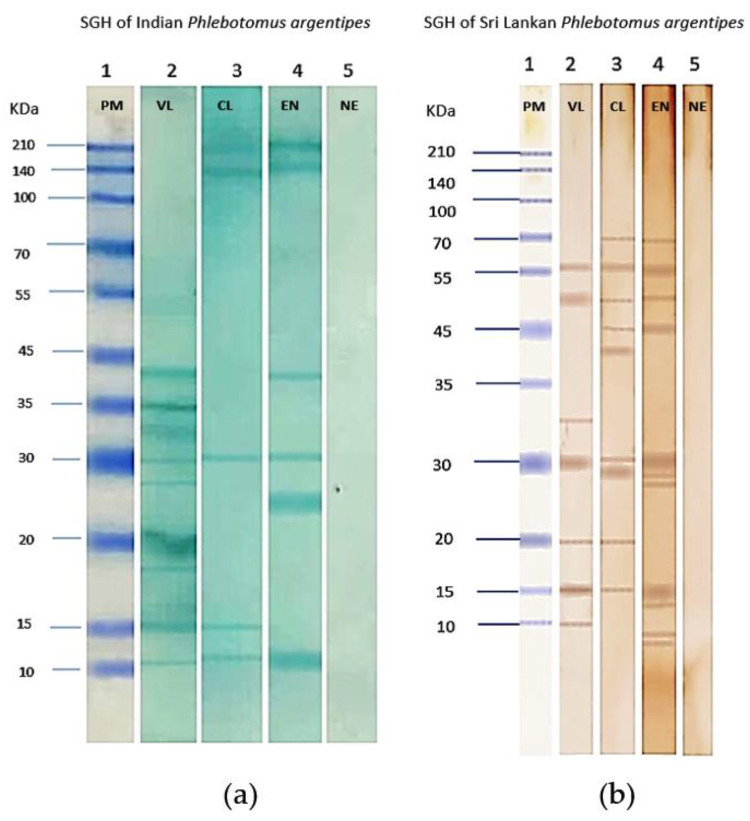
Western blots indicating human IgG serum reactivity with salivary gland homogenate (SGH) of *Phlebotomus argentipes.* (**a**) SGH of *Ph. argentipes* of Kerala, India. (**b**) SGH of *Ph. argentipes* of Sri Lanka. Lane 1: pre-stained protein marker (PM); Lane 2: serum of visceral leishmaniasis patient (VL); Lane 3: serum of cutaneous leishmaniasis patient (CL); Lane 4: serum of endemic individuals (EN); Lane 5: serum of non-endemic individuals (NE).

In our Western blot analysis of saliva samples, we observed a strong immunogenic response. In Indian *Ph. argentipes* SGH (Figure 7a), distinct bands were detected between ~10 kDa and ~40 kDa. Conversely, in Sri Lankan *Ph. argentipes* SGH (Figure 7b), bands ranged from >10 kDa to 70 kDa. Serum from a VL individual in India showed nine immunogenic bands with molecular weights (MW) ranging from ~10 kDa to ~40 kDa. In contrast, serum from a CL individual showed only three bands at ~10 kDa, ~15 kDa, and ~30 kDa (Figure 7a), while an endemic individual’s serum reacted with four immunogenic bands at ~10 kDa, ~25 kDa, ~30 kDa, and ~40 kDa. Notably, common immunogenic bands were identified across all, with bands at ~10 kDa and ~30 kDa showing reactivity in VL, CL, and endemic individuals from India.

When exposed to the SGH of Sri Lankan *Ph. argentipes*, both VL and CL sera displayed broad reactivity. VL serum showed immunogenicity, with six bands ranging in molecular weight from ~10 kDa to ~55 kDa, while CL serum reacted and showed nine immunogenic bands with MW ranging from ~15 KDa to ~70 kDa. Endemic serum exhibited reactivity with nine immunogenic bands, similar to CL serum, with MW ranging from >10 kDa to 70 kDa. Common immunogenic bands were observed at ~15 kDa and ~30 kDa with the tested sera of VL, CL, and endemic individuals (Figure 7b). 

The 30 kDa immunogenic band was consistently present in both Indian and Sri Lankan sera across two sets of SGHs (Figure 7), indicating antibody development in endemic individuals targeting specific salivary proteins. Sera from NE individuals showed no immune response against *Ph. argentipes* salivary gland proteins (Figure 7).

### 3.7. Proteomics Analysis

The most immunogenic protein band (size approximately 30 kDa) observed across two sets of SGH from Indian and Sri Lankan *Ph. argentipes* was selected for proteomic identification. The identified proteins, PagSP05, belong to the antigen 5-related protein family, PagSP06 belongs to the PpSP32-like proteins family, and PagSP17 is a novel protein with a molecular weight of 29 kDa. Its function is yet unknown. These proteins exhibit significant similarity to salivary proteins documented in *Ph. argentipes*, as confirmed by data retrieved from the NCBI GenBank database (Table 1).

## 4. Discussion

Our study compared the morphology and molecular characteristics of *Ph. argentipes* isolated from study sites in India and Sri Lanka, alongside an analysis of its immunogenic salivary proteins. Over the past decade, these regions have experienced a high incidence of leishmaniasis cases, primarily caused by the parasite *L. donovani*. This parasite exhibits phenotypic differences and is transmitted by the vector *Ph. argentipes*. This study represents the first comparative analysis of these two countries in this context. 

Our study focused on collecting sandflies during their peak activity periods in both India and Sri Lanka. These targeted collection periods were selected to maximize the capture of sandflies during their most abundant and active stages. This approach ensured a robust dataset for understanding the ecological dynamics of sandfly populations and their potential implications for the epidemiology of CL in the studied regions.

In our investigation, we noted morphological similarities among *Ph. argentipes* flies collected from study sites in both India and Sri Lanka. A prominent characteristic is the observation that the lengths of the sensilla chaetica, in relation to the second antennal flagellomere, exceed a ratio of 0.5 in female specimens of both Indian and Sri Lankan populations [18,19]. *Ph. argentipes* is known to comprise three complex members: *Ph. argentipes s.s., Ph. annandalei*, and *Ph. glaucus* [9,18,19]. Taxonomical studies have provided evidence for the presence of *Ph. glaucus* in the Southern parts of India, including Kerala, and Sri Lanka, similar to what we observed in our study [18,19]. Additionally, distinctive features, such as the shape of the spermatheca in female flies and the presence of a three-lobed paramere in male flies were observed, further reinforcing the finding of shared characteristics between the Indian and Sri Lankan sandfly populations. 

We conducted a comprehensive phylogenetic analysis, providing new and valuable data for the underrepresented Sri Lankan sandfly populations in the selected study sites while effectively utilizing the available Indian sequences for comparison. The phylogenetic analysis uncovered genetic divergence between sandflies from India and Sri Lanka, segregating them into two distinct clades (clades A and B). However, a study reported that sibling species A and species B of *Ph. argentipes* in Sri Lanka [32,33] showed no genetic variation, except for differences in the ratio between sensilla chaetica and the second antennal flagellomere, indicating that the morphological differences that were observed do not correspond to the genetic differences. In the phylogenetic tree, we observed a mixture of clades between *Ph. argentipes* sandfly sequences from Kerala, India, and Bihar, India. Notably, these two states are geographically distant (around 2000 km), with Bihar located in north India and Kerala in the south. However, Kerala primarily reports a mixture of cases of both CL and VL, whereas Bihar is a hotspot for VL [34]. In a recent genetic study, *Ph. argentipes* specimens from Kerala and Sri Lanka formed a single cluster for the *cox1* gene [35]. However, in our investigation, we identified nine synonymous mutations in the *cox1* gene sequences of Sri Lankan *Ph. argentipes* in comparison to the *Ph. argentipes* from both Kerala and Bihar, India. These synonymous mutations, which do not alter the amino acid sequence of the encoded protein, are often referred to as “silent” mutations [36]. Consequently, the proteins, including salivary antigens and other functional proteins produced by both sandfly populations, may share similarities. This could support the observed similarities in our salivary protein profiles. 

The immunogenic salivary proteins from both the Indian and Sri Lankan *Ph. argentipes* showed a consistent and robust immunogenic response to the tested sera. We observed some differences in the immunogenic profiles of *Ph. argentipes* from both countries. The composition of sandfly saliva varies not just among different species [21,22] but also among populations from different geographical regions [26,27,37,38]. A study by Hosseini-Vasoukolaei et al., 2016 [39], also revealed the differential expression profiles of two salivary proteins within the same species of *Ph. papatasi*, emphasizing the complexity of these expression patterns.

The VL sera exhibited more immunogenic bands compared to the other sera tested in the study, which may be due to the presence of high levels of antibodies [40,41]. The immunogenic band around 30 kDa consistently appeared in the sera from both Indian and Sri Lankan subjects, being common to the tested sera of VL, CL, and endemic individuals. Moreover, this salivary protein shows a promising ability to discriminate individuals with *Ph. argentipes* exposure from those without exposure. 

In the ESI analysis of the mass spectrometry data of the 30 kDa band of Indian *Ph. argentipes,* the band was found to be similar to the abundant proteins described in the Edman degradation of Anderson et al., 2006: PagSP05, PagSP06, and PagSP17 [42,43]. This observation suggests that one or more of these proteins might play a crucial role in eliciting an anti-salivary antibody response against SGH. An investigation evaluating recombinant forms (rPagSP04, rPagSP05, and rPagSP06) for their efficacy in ELISA and immunoblot assays demonstrated that the PpSP32-like recombinant PagSP06 emerged as the most promising antigen, correlating most strongly with the IgG response to SGH of *Ph. argentipes* in Bangladesh [44]. However, the functions of the PpSP32-like protein are still unclear [26,27]. This protein family was recognized as a significant target of the antibody response triggered by bites from *Ph. papatasi* in humans in the study conducted in Tunisia [45,46]. The study based on yellow-related protein rPagSP04 and antigen 5-related protein rPagSP05 found that these antigens were unsuitable for measuring *Ph. argentipes* exposure [44]. Iniguez et al. [43] validated a combined recombinant salivary antigen comprising rPagSP02 and rPagSP06. Using sera from individuals in endemic regions, they successfully demonstrated the efficacy of this composite antigen in an Indian context, where VL is widespread. Unfortunately, the details and function of PagSP17 in the ESI analysis are not well-known, as it is identified as a novel protein [42]. 

The salivary protein with a size of ∼15 kDa (PagSP02- PpSP15-like proteins) is the second most common protein, and exhibited immunogenic reactions with two sets of SGHs from India and Sri Lanka. A separate study [47] experimentally confirmed the immunogenicity of this particular ∼15 kDa salivary protein in research conducted in India using mice, and the identified immunogenic proteins were identical to the previously reported protein of SP15 family of *Ph. argentipes* of the NIH colony. Consequently, PagSP02 holds potential as playing a biomarker role alongside PagSP06 [47]. 

The present study was limited, as it considered only one serum category for immunogenic profiles in the Western blot analysis, thereby reducing the exploration of antigenic variations and the breadth of exposure to sandfly vectors. We recognize this as a significant limitation and emphasize that incorporating multiple donors from endemic sites of leishmaniasis would improve the understanding of the immunogenic profile of salivary proteins. Such an approach would provide a more comprehensive assessment and yield insights into the variability of immune responses within affected populations. Environmental factors such as the temperature, the humidity, and the blood-feeding history of the sandfly can affect the expression of salivary proteins. However, this study was unable to account for changes in these factors over time.

## 5. Conclusions

In conclusion, our comprehensive investigation of *Ph. argentipes* in South India and Sri Lanka has unveiled significant insights, revealing key differences at the morphological, molecular, and salivary protein levels and highlighting the need for a nuanced understanding of sandfly populations. A comparative analysis of the immunogenicity of salivary proteins from *Ph. argentipes* in India and Sri Lanka indicates a high level of similarity. This suggests that these proteins could serve as common targets for markers of vector exposure or stimulants for cellular immune responses in humans across various geographical regions. Expanding research to validate the efficacy of recombinant antigens, such as PagSP02 and PagSP06, in a large cohort would open promising avenues not only for the development of targeted tools in leishmaniasis prevention and control, but also for understanding infection dynamics. Leishmaniasis, a challenging zoonosis to diagnose due to its nonspecific symptoms, spreads through sandflies and asymptomatic mammalian reservoirs. In-depth vector studies are crucial. By targeting shared immunogenic proteins in *Ph. argentipes*, we can develop diagnostic tools for exposure detection and vaccines to reduce transmission and improve control measures. Our findings emphasize the importance of focusing on common immunogenic salivary proteins for the development of new interventions. Specifically, these proteins can be used to create diagnostic tools that help identify exposure to sandfly bites, improving surveillance efforts. By targeting these shared proteins, we can create broad-spectrum interventions applicable across different geographical regions. Overall, our findings contribute valuable information to the ongoing efforts aimed at addressing the challenges posed by leishmaniasis in the ISC.

## Figures and Tables

**Table 1:** Identification of 30 kDa protein band excised from the SDS-PAGE gel.

Features	Protein Identified/Sequence Name		
	SP17	SP06	SP05
Molecular weight of the protein/KDa	31.702	27.000	32.357
Total number of peptides matched to each protein	6	6	16
Number of unique peptides matched to each protein	6	6	16
Confidence score	34.662	30.948	90.228
Peptide sequences	NVVNDIVEVTFGDEEPLN TGVFIVK GFNNSNYIK VNYNLYREELEQK YDYNTNK DILQLQGFIR	LFETNKAEEVIAK DFPVPTADEK ELVIDESTAR DAPATYVFK VRQNSQDR	LRDR LAEYNVR WNDELAK EWFLEYK GSMSPFQSAAK DAQLMPITEDTKK LCTFGPGLPARPHIGCK AIGHFTAFIHEK
Best match to protein database	29 kDa salivary protein PagSP17 of *Phlebotomus argentipes*	25 kDa salivary protein PagSP06 of *Phlebotomus argentipes*	29 kDa salivary antigen 5-related protein PagSP05 of *Phlebotomus argentipes*
Best match UniProtKB accession #	Q0ZST4	Q0ZSU3	Q0ZSU4
Best match Gen Bank accession #	ABA12147	ABA12138	ABA12137

kDa: Kilo Daltons; UniProtKB: The Universal Protein Resource; #: number.

## Data Availability

The original contributions presented in the study are included in the article/Supplementary Materials, further inquiries can be directed to the corresponding author.

## Supplementary Materials

The following supporting information can be downloaded at https://www.mdpi.com/article/10.3390/microorganisms12071459/s1, Figure S1: Agarose gel electrophoresis. PCR products of sandfly specimens collected in Ambalantota, Sri Lanka for COI marker. Lane 1: 100 bp DNA ladder; Lanes 2–13: PCR products; Lane 14: positive control. Table S1: Overall number of female sandflies captured during the field collections.
