# Supplementary material for: Comparative Analysis of Phlebotomus argentipes Vector of Leishmaniasis in India and Sri Lanka
Source: Microorganisms. 2024 Jul 18;12(7):1459. doi: 10.3390/microorganisms12071459 (PMC11278908; doi:10.3390/microorganisms12071459)
Supplement: Supplementary file 1 [file microorganisms-12-01459-s001.zip › microorganisms-3070627-supplementary.pdf]

Table S1. Overall number of female sand flies captured during the field collections

	Collection in outdoor bathrooms				Collection near cattle sheds			
**Sand fly species**	**NF**	**BF**	**Gravid**	**Total**	**NF**	**BF**	**Gravid**	**Total**
Phlebotomus argentipes	02	03	03	08	147	71	38	256
Phlebotomus colabaensis	02	0	01	03	10	12	11	33
Sergentomyia himalayensis	18	07	13	38	14	03	03	20
Sergentomyia babu	37	06	08	51	02	0	01	03
Segentomyai dhandai	01	01	02	04	01	0	01	02
Sergentomyia bagdhadis	04	02	02	08	03	0	0	03
**Total**	**64**	**19**	**29**	**112**	**175**	**86**	**53**	**314**

NF: non fed, BF: blood fed

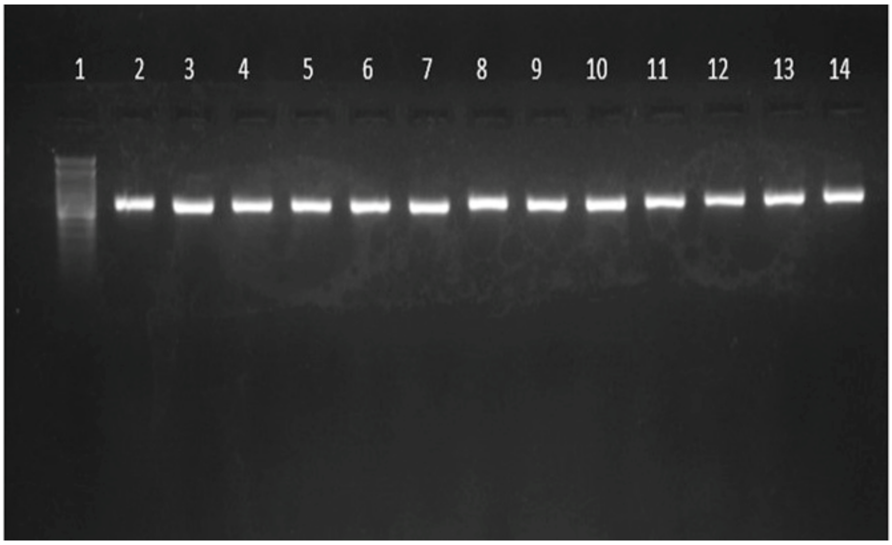

Figure S1: Agarose gel electrophoresis. PCR products of sandfly specimens collected in Ambalantota, Sri Lanka for COI marker. Lane 1: 100 bp DNA ladder; Lanes 2–13: PCR products; Lane 14: positive control.
